# Leading top-down implementation processes: a qualitative study on the role of managers

**DOI:** 10.1186/s12913-018-3360-y

**Published:** 2018-07-18

**Authors:** Håkan Uvhagen, Henna Hasson, Johan Hansson, Mia von Knorring

**Affiliations:** 1 Department of Learning, Informatics, Management and Ethics (LIME), Medical Management Centre (MMC), Karolinska Institutet, 171 77 Stockholm, Sweden; 2 Research and Development Unit for Elderly Persons (FOU nu) Stockholm County Council, Stockholm, Sweden; 3 Department of Public Health Analysis and Data Management, Public Health Agency of Sweden, 171 82 Solna, Sweden

**Keywords:** Managerial role taking, Implementation, Primary healthcare, Academia, Qualitative study

## Abstract

**Background:**

Leadership has been identified as an influential factor in implementation processes in healthcare organizations. However, the processes through which leaders affect implementation outcomes are largely unknown. The purpose of this study is to analyse how managers interpret and make sense of a large scale top-down implementation initiative and what implications this has for the implementation process. This was studied at the implementation of an academic primary healthcare initiative covering 210 primary healthcare centres in central Sweden. The aim of the initiative was to integrate research and education into regular primary healthcare services.

**Methods:**

The study builds on 16 in-depth individual semi-structured interviews with all managers (*n* = 8) who had operative responsibility for the implementation. Each manager was interviewed twice during the initial phase of the implementation. Data were analysed using a thematic approach guided by theory on managerial role taking based on the Transforming Experience Framework.

**Results:**

How the managers interpreted and made sense of the implementation task built on three factors: how they perceived the different parts of the initiative, how they perceived themselves in relation to these parts, and the resources available for the initiative. Based on how they combined these three factors the managers chose to integrate or separate the different parts of the initiative in their management of the implementation process.

**Conclusions:**

This research emphasizes that managers in healthcare seem to have a substantial impact on how and to what extent different tasks are addressed and prioritized in top-down implementation processes. This has policy implications. To achieve intended implementation outcomes, the authors recognize the necessity of an early and on-going dialogue about how the implementation is perceived by the managers responsible for the implementation.

**Electronic supplementary material:**

The online version of this article (10.1186/s12913-018-3360-y) contains supplementary material, which is available to authorized users.

## Background

An aging population with more chronic diseases and complex conditions [1] increases the pressure on healthcare systems to continuously improve quality and implement new evidence-based methods [2]. To deal with this situation, healthcare organizations need to be increasingly innovative and implement new ways of organizing the manner in which they deliver the care that is provided [3]. However, implementation processes have been described as unpredictable and complex [4–7] where several factors, in addition to the content of change, affect the outcomes [8–10]. Leadership has been identified as one influential factor in implementation processes [4–7, 9, 11–14].

In a study of leadership in implementation of evidence-based methods in healthcare, Aarons et al. [15] suggested a comprehensive model including four aspects that managers need to cover in order to achieve effective implementation processes. First, they need to be proactive by producing and communicating a plan for the implementation, and by finding and addressing circumstances that hinder the implementation process. Second, they must have knowledge and understanding of implementation issues and be able to answer staff questions about the implementation. Third, they should appreciate employee implementation efforts, give feedback, and support staff members in learning more about the implementation. Fourth, they need to be perseverant and reactive, and continuously address various challenges as they arise throughout the implementation process [15]. Øvretveit [3, 16] has emphasized the responsibility of managers to secure necessary conditions in the context in which an implementation takes place, for example, to provide adequate competence, time, and financial resources for the implementation process. This function was also identified as an important managerial task in a literature review by Gifford et al. [17].

Nevertheless, although the relevance of leadership in implementation science has been increasingly acknowledged in recent years, the processes through which leaders can affect implementation outcomes in healthcare are still largely unknown [3, 7, 12, 15, 18–21]. The present study looks further into this issue and explores how healthcare managers take their role in a large-scale implementation initiative. The specific aim of this study is to analyse how managers interpret and make sense of a large scale top-down implementation initiative and what implications this has for the implementation process.

### Theoretical starting point

**Fig. 1:**
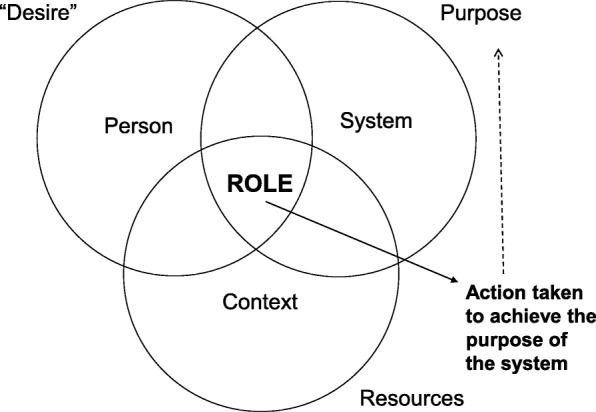
Model of managerial role taking based on the Transforming Experience Framework (TEF) [22, 23]

Here, a theory on managerial role taking based on the Transforming Experience Framework (TEF) [22–25] is used as a starting point for the line of reasoning. With its strong focus on the importance of a well-communicated purpose for actions taken within organizational contexts, the TEF provides a useful model for understanding how managers take their role and influence implementation processes. This framework is based on the original assumption by Reed [25] that persons’ role taking (i.e. how they handle their formal functions and act within organizational contexts) [24, 26] is geared by how they perceive the purpose of that specific system. Building on the TEF, the way that managers take their role can be described as emanating from the three aspects of person, system, and context (Fig. 1). *Person* deals with how individuals handle themselves (e.g. regarding their own feelings, expectations, and desires) as a “person in role”. *System* refers to how individuals perceive and understand the purpose of the organization or system in which they are supposed to act as a manager “in role”. *Context* is related to how individuals perceive and handle the context and the resources within the system [22, 24]. This picture indicates possible tension between personal needs and desires and the purpose of the system in which the individuals take on their professional roles [24]. In this way, Reed [25] describes that a role in not passively adopted to, but something actively taken by identifying the purpose of the system the individual belongs to and acts in, gaining ownership of that purpose, and choosing how to behave and what actions to take to accomplish the purpose of the system [25].

## Methods

### Design

Due to lack of empirical studies on how managers take their role in large-scale implementation initiatives the study used an exploratory design drawing on data from qualitative interviews [27].

### Empirical setting for the study

The Academic Primary Healthcare (APH) initiative is used as empirical setting. The initiative was introduced by policy makers and the senior management team in Stockholm County primary care in 2011. It was further developed and managed together with public and private primary healthcare providers, the medical university, and the three medical university colleges in the county. Staff recruitment and retention, and also a desire to improve the quality of primary healthcare, were described as key drivers of the initiative [28, 29]. The aim was to achieve closer integration between academia and primary healthcare practice by promoting and coordinating clinical training for students, increasing and stimulating research in healthcare practice, implementing research findings and conducting quality improvement interventions, and enhancing prerequisites for clinical research education [28]. The initiative was also considered to aid the on-going general transfer of services from emergency hospitals to primary healthcare [29].

**Fig. 2:**
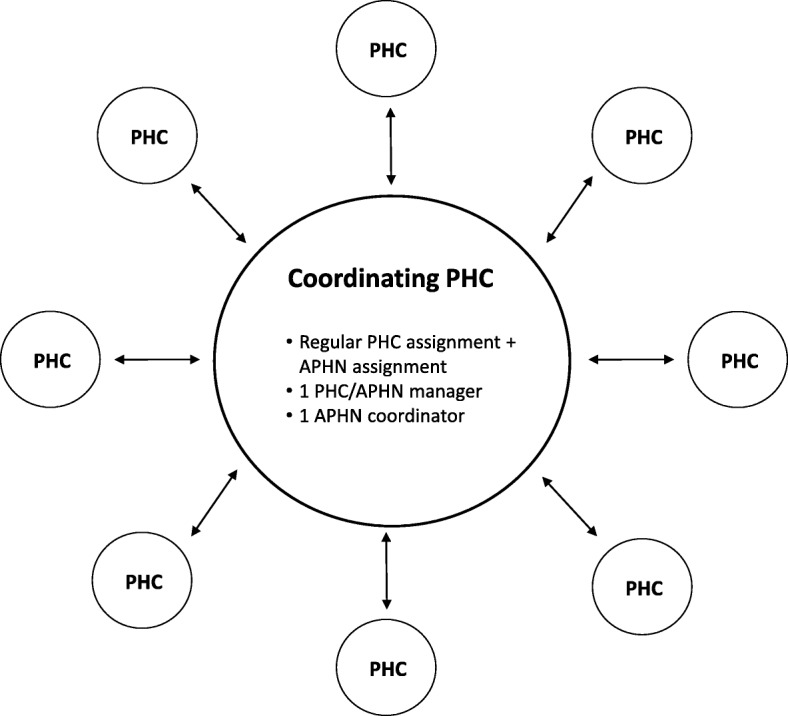
A schematic layout of an Academic Primary Healthcare Network (APHN). In reality 20–40 PHC that are closely located geographically form an APHN. Eight such APHNs are established in the region covering all public and private PHC

The initiative was implemented in the whole county, covering 210 public and private primary healthcare centres (PHCs). All PHCs were invited to apply to become a coordinating centre, and, after an application process, four coordinating centres were launched in 2011 and another four in mid 2014. The managers of the coordinating centres were given the responsibility of leading and coordinating the implementation of the APH initiative that included all PHCs in their geographical area. Eight APH Networks (APHNs) were formed in this way, each comprising from 20 to 40 PHCs. After becoming APH coordinating centres, the eight PHCs continued to handle their regular healthcare assignments, with the addition of the manager now also being accountable for performance of the APHN. A schematic layout of the APHN structure is presented in Fig. 2.

The initiative stipulated financial compensation for a part-time coordinator at each of the coordinating centres. All coordinators were required to have a doctoral degree, and they were affiliated with the regional medical university but were employed and supervised by the manager at the APH coordinating centre. The task of the coordinators was to promote, coordinate, and improve education and research in their APHN in collaboration with the universities and primary healthcare staff.

### Participants and data collection

All eight managers at the coordinating APH centres were interviewed individually twice, in total 16 interviews. The first interview round (*n* = 8) focused on the following: the background and concept of the APHNs; expectations and experiences from similar initiatives; actions taken to implement the APHNs; intermediate outcomes and the future APHN activities. The second interview round (*n* = 8) focused more specifically on the manager role in the implementation process (see Additional file 1). For the managers at the four APHNs that were launched in 2014, both interviews rounds were conducted on the same occasion. Thus the time elapsed between the two interviews differed for the participating managers, ranging from being either concurrent (*n* = 4) or up to 12 months apart (*n* = 4).

The managers were invited via email to schedule a time and date for an interview. The email described the research project and its objectives, and also stated that the participating managers had the right to withdraw from the study at any time. Audio-recorded informed consent to join the study was obtained from all participants. The 16 interviews were conducted between October 2013 and December 2014. Fourteen of the interviews were carried out face-to-face, and the remaining two were conducted by telephone for practical reasons. The interviews lasted between 32 and 57 min, and they were audio-recorded and transcribed verbatim (using codes rather than names to protect confidentiality), resulting in approximately 85,000 words of data.

Four of the managers were women, and four were men. Four had a doctoral degree or were enrolled in PhD programmes at the time of the interviews. Four were physicians, and the other managers represented other healthcare professions.

### Data analysis

To obtain a rich material, the two different data-sets were merged into one data-set for analysis. A thematic approach [30] was used to analyse data. Initial familiarization with the entire data set was achieved by reading and re-reading the transcribed interviews several times, after which statements related to the managerial role were identified throughout the data set, strictly guided by the main aspects of the TEF model [23]. By applying this deductive procedure, all statements that were in any respect related to issues of *context*, *person*, *system*, or *purpose* (as conceptualized in the TEF model) were extracted to provide the unit of analysis.

Parallel to the initial deductive procedure, and continuously throughout the entire analysis, memos with initial ideas for potential themes and the overall story related in the data were recorded in writing and discussed by the first and last author.

In the next inductive step, all statements identified in the previous step were coded, and preliminary themes in the data were identified. A thematic map relating the identified themes to each other was generated and refined in an iterative process going “back and forth” between empirical data and preliminary themes in the thematic map. At this stage, the second and third author read interview transcripts to confirm that the identified themes and the relationships between them were grounded in the data. Finally, refinements and specifications of themes were completed based on in-depth discussion and negotiated consensus [31] between the first and last author, and relationships between the themes were developed.

In the following section, quotations that richly exemplify the identified themes are used to illustrate the findings. Each quote is identified by a letter that designates a specific interviewee. In the transcript excerpts, the symbol /…/ indicates an omission, and [] indicates an addition; these changes have not altered the meaning of the quotations and were made solely to enhance readability or secure anonymity of the participants. All the presented quotes have been translated from Swedish.

## Results

The analysis showed that the managers gave rather coherent descriptions of how the aim of the implementation was formulated in the original policy documents. They also had rather similar understandings of why the initiative was necessary, for example, referring to the need for recruitment and for raising the status and quality of primary healthcare services. One manager explained it like this:

*[The initiative] increases both the status and the quality of primary care. /…/ the connection with evidence and knowledge and research and the like was previously largely secondary and ignored. This made all the other [actors] in the care system to some extent think that primary care doesn’t really take its responsibilities seriously. (A).*

Another manager put it like this:

*Primary healthcare has no tradition of research and education. /…/ And maybe more people will be interested in working in primary care, because, if you’re going to do research, [it] will have to be done out here [in primary healthcare] where the patients are. (H).*

### Factors influencing how the managers interpreted the implementation task

Although the managers were fairly similar regarding their descriptions of the content and the rationale of the initiative, they differed with respect to how they interpreted and made sense of the task. That is, it seemed that their efforts to integrate research and education in healthcare practice were based on the combination of three factors, which are described in detail below.

#### The perception of the different parts of the initiative and their interrelationship

The manner in which the implementation task was interpreted was based on how the managers perceived the different parts of the initiative (i.e. academia and primary healthcare practice) and the relationship between them. The managers described these aspects in two different ways. One manager said the following:

*Much of what is part of being an APHN represents things that we’re already doing. And they’re kind of integrated in the whole and are therefore already part of the job. Then that might take up a little more space and become a somewhat larger part of things and expand. But in a way /…/ there’s kind of no difference like from zero to one, no it’s more like different degrees. (E).*

In this example, the manager describes a close relationship between the different parts of the initiative, depicting them as “integrated in the whole”, and argues that they are to a large extent already “included in the job”.

Another way of describing the relationship between the parts of the initiative was instead to emphasize their disparities. One manager made this statement:

*I see that one of the major problems affecting me, is that I have two assignments that have to be balanced against each other and are sometimes almost conflicting. (D).*

For this manager, the challenge of the implementation was to combine what was perceived as two different tasks. Hence the relationship between the parts of the APH initiative was interpreted as being inconsistent and entailing tasks that have to be weighed against each other.

Another manager emphasized academia and practice as being different and contrasting when describing the APHN task:

*It’s more like that APH is more dynamic compared to regular primary healthcare. /…/ That’s what APH is, you’re supposed to develop and dare to try different things. (F).*

The same manager continued:

*It’s largely about helping yourself to things and seeing what happens, that is, not following guidelines or the like, but instead actively participating in development, teaching, research, be curious, start challenging things, and then strive systematically to develop primary care. (F).*

In these statements, the manager implied that academia and practice differ in nature to some degree. The APHN assignment was labelled as being “more dynamic” and including “testing new things”, “curiosity”, “development” and “starting to challenge things” in contrast to what was perceived as things that are included in the ordinary assignment of providing primary healthcare.

Hence the managers perceived the various parts of the initiative and the relationship between them in two different ways: as cohesive or as dissimilar and detached.

#### The managers self-perception in relation to the different parts of the initiative

Another factor identified as having impact on how the managers interpreted the APH initiative was associated with how the managers perceived themselves in relation to the different parts of the initiative.

The analysis showed that the managers used two significantly different approaches to position themselves as managers in relation to the different parts of the initiative depending on whether or not they regarded themselves as academics. All of the participating managers had university college training, but they nonetheless considered the state of being an academic in terms of having or not having a PhD degree.

Having academic experience, in this case having a PhD, was described as important in the implementation of APH. One manager formulated it like this:

*I can handle the jargon and I comprehend, I feel fairly at ease in academic contexts. But there are a lot of managers that don’t have that background, and I think that makes it very difficult. (E).*

In this example, the manager (who has a PhD) emphasizes having knowledge of “the academic lingo” and familiarity with the academic settings, and also points out that many mangers “don’t have that background”, which makes the implementation task “very difficult”.

Statements related to being a “non-academic” were also made by the managers that did not have a PhD degree. One manager said the following:

*I feel like I lack some degree of academic skills. The healthcare centre is more hands-on, so to speak, I have been employed [in primary healthcare] for many years myself and sort of know how to handle these things. The APHN is new for me, and I have experienced a lot of things that make me feel like I don’t really have the academic background for this. It’s been hard. Sometimes I’ve felt that it’s kind of a little like a minor culture clash between two worlds. (D).*

In this statement, the manager describes a lack of research experience and a higher academic degree that makes it difficult to combine parts and to handle the “cultural clash” between the “two worlds”.

Another manager expressed it like this:

*I don’t have a PhD. /…/ At meetings and such, I’ve been hearing some talk about how in the future it might be best to have a manager with a PhD. But I’m not so sure that that’s right. Because if you’ve also been in that world too long, you can be influenced by it. Because we also have a reality. And I’m not so sure that my performance as a manager is necessarily less satisfactory because I don’t have a PhD. (B).*

This manager also highlight lack of research experience and a higher academic degree but reflected on whether this is an important aspect, and also considered the risk of being “too academic” and “too distant from healthcare reality” in the role as a manager to allow the integration of academia and practice.

#### The perception of available resources for implementation of the initiative

A third factor identified as having impact on how the managers interpreted the implementation task was related to how they perceived the resources that were available for the initiative. One manager formulated it like this:

*You’re supposed to be productive.* /…/ *And then there’s been a feeling that we should also take on students. Yeah, and then they come, and you’re supposed to do what can seem like two things at once: you’re supposed to be a good supervisor who is supposed to deal with things calmly and at the same time be productive.* /…/ *It doesn’t make sense. (B).*

Here, the manager describes the challenge of performing two different tasks at the same time. The perception of difficulties in both “producing healthcare” and “finding the time to facilitate clinical training” makes the manager describe the implementation as a “mismatch”.

Another manager illustrated this by questioning the sustainability of the initiative in relation to initiative implementation resources:

*I think you need to be honest and kind of reconcile with those who give us the task* /…/ *because this is definitely difficult to maintain in the long run. What are your goals? What do you want? What is in the overall picture? We have to settle this once and for all. Because managers out there will be disappointed if the whole thing is, sort of, perceived as ‘what’s the point’? (G).*

Considering this statement, the perception of there being limited resources even makes this manager question the overall purpose of the initiative and links this to “disappointment” and “hopelessness” among participating healthcare managers.

### Implications for the implementation process

The three factors described above seem to have implications for how the managers handled the implementation process. Two different approaches were identified: an integrating approach and a separating approach.

The integrating approach was taken by the managers when the implementation parts were perceived as cohesive, and when available resources for implementation were considered to facilitate, or at least not hinder, integration. The following statement illustrates an integrating approach, where the manager describes the parts as “being intertwined” and the role as an academic and healthcare manager as not being contradictory:

*But for my part, there are no difficulties, because I think that this turns up again in other questions regarding development, if you’re interested in development issues and research, then I think there are several areas in which I have worked in a similar manner,* /…/ *So it’s kind of like two roles, but I don’t think that they’re opposing roles. (F).*

The separating approach was taken when the parts of the initiative were perceived as contradictory and/or when available resources were considered as counteracting an integrated implementation. The following statement emphasizes the relationship between the parts as “being counteractive”, connecting this to a perception of limited implementation resources that hamper an integrating approach and thus force separation and prioritization of the parts of the initiative.

*But if you want to summarize this in a single word that represents what is definitely the major problem, the word is ‘time’, which can also be translated into money, of course, but that’s what it’s all about. If colleagues could feel like it was possible to participate in various activities, then I think that that’s what they would do. And get many who are really interested to do the same. But this contradicts the other goals that we’re supposed to achieve (D)*.

The separating approach was enhanced when the managers considered the integration of the tasks to constitute a threat to performance of the part of the initiative that was perceived as having the highest priority. In the following comment, the manager discusses how to combine the tasks in the initiative but ends up choosing to prioritize and protect the regular healthcare practice assignment—the delivery of care to patients:

*Well you know it involves /…/ training activities for example at the APHN. But if there’s illness that has to be dealt with here at the healthcare centre, then we just have to tell those who have signed up that no, unfortunately that’s not possible today, you can’t participate in that particular training. So that’s the way that I prioritize care offered to patients. (D).*

The perception of it being difficult to combine the parts of the initiative was experienced as a real dilemma by the managers. The following statement describes a feeling of nearly being trapped between an integrated and a separated approach. This manager had the ambition to combine the parts but at the same time found it difficult to motivate decisions, both for him−/herself and internally within the organization.

*That’s a difficult part of my role, telling them /…/ that now you have to meet 60 patients per week, and at the same time saying that on some other occasion or the day before or the day after that I want you to participate in this activity /…/ and be away from work for two hours or a half day. Aha, they say, but how does that affect my output? /…/ So that doesn’t tie in either. /…/ obviously I feel divided, and it’s extremely difficult. At the same time, I have to ignore it. (C)*.

## Discussion

The aim of this study was to analyse how managers interpret and make sense of a large scale top-down implementation initiative and what implications this had for the implementation process. The results show that even though the primary healthcare managers studied here were given the same implementation assignment, and they provided coherent descriptions of why the initiative was needed, they managed the implementation process differently. The way the managers interpreted and made sense of the task was influenced by three crucial factors: how these individuals perceived the different parts of the initiative, how they perceived themselves in relation to these parts, and how they perceived the resources available for the initiative. These factors had implications for the implementation process, for which two approaches were identified: an integrating and a separating approach.

The two approaches identified in this study are similar to the findings of a study by Dellve and Wikström [32] focused on healthcare managers’ approaches to managerial and professional logics in healthcare, which showed that the managers perceived such logics as either contradictory or consistent.

When attempting to integrate research and education in primary healthcare practice the managers in the present study positioned themselves as academics or non-academics. This implies that perceptions of who you are and where you belong exist and are challenged in implementation processes. The same line of reasoning has been offered by Brown [33] and Langley et al. [34] in reports indicating that aspects of identity are intensified in a context characterized by tensions, constraints, or changes in work procedures.

The current results also indicated that the managers’ perceptions regarding the availability of initiative implementation resources had implications for how the managers handled the implementation process, which confirms a previous report outlining the role of resources in such actions [35]. It seems that the implementation task is hampered by perceptions of limited resources activated by the mismatch of resources that seems to arise when the parts of an implementation are combined. This conclusion also agrees with data reported by Kitson and Harvey [36] and Reichenpfader et al. [12] emphasizing the importance of adequate resources in implementation processes.

Taken together, the findings suggest that the managers’ perceptions of the different parts of the implementation initiative, of themselves in relation to these parts, and of the resources that are available for the initiative have an impact on how the task is constructed and thereby also on the actions that are taken in the implementation process. This observation, in agreement with what has previously been illustrated in the TEF model [23], highlights the importance of having an ongoing dialogue about how the different actors in a system perceive the overarching purpose of implementation initiatives and the resources available to achieve that purpose [22–24]. It seems that the managers in the present study actively took into consideration the purpose of the implementation in a way that influenced the manner in which the implementation process was handled, in addition to what was actually stated in the written description of the implementation task. This indicates that it is not pre-determined how an implementation will be managed or how things will develop. Consequently, caution should be observed when carrying out an implementation process that relies exclusively on written assignments as a means of directing the implementation and its objectives.

One interpretation of the current results is that the managers integrated or separated the parts of the initiative to move away from what was perceived as a diffuse managerial role. In other words, by integrating or separating the parts, the managers themselves constructed role clarity through their perceptions of the parts. This has previously been described in terms of sense making [37]. By constructing the parts as possible or not possible to combine, it appears that the managers were able to adopt the role as sense makers and thereby address the need “to bring order to what has become unclear or chaotic” ([38], p., 365) in relation to the APHN assignment.

In the present study, separation and prioritization were also found to be enhanced when the combination of the parts was perceived as a threat to performance related to the part with the highest priority. This illustrates a dynamic approach to choosing integration or separation. It seems that managers adhere to the combination of the parts until the point where perceived advantages of the combination fall below perceived risks of not performing in relation to the part with highest priority. Hence it seems that integrating or separating implementation parts enables the managers to protect the part with highest priority.

This picture confirms that implementation in healthcare practice is not a straightforward process [9] and also that it is not the content of the implementation as such that influences the implementation and its outcomes, but rather how these parts are perceived by the managers [10].

### Methodological considerations

A strength of this study is that all managers at the coordinating centres in the APHN initiative were included which makes it a total investigation [39]. Furthermore, all the authors read the transcripts and helped identify themes and the relationships between them, which strengthens the internal validity of the results. In addition, the first author, who conducted the interviews, was highly familiar with the operations of primary healthcare, and this contributed to the use of informed follow-up questions in the interviews.

The method used for analysis in this study did not allow for analysing any potential differences in self-perception between the managers based on profession. However, that does not mean that there are no differences. This needs to be explored in further studies.

In this study we used the TEF model as a starting point for the analysis. Through its focus on the importance of purpose for how actions are taken within organisations, the model provides a useful tool for understanding how managers take their role and influence implementation processes. The use of the model to identify the unit of analysis made deeper analysis of the data possible and aided identification of aspects that otherwise might have been missed. In the study we only used the TEF model in the first step of the analysis, however our preunderstanding of the concepts in the model may of course have influenced our general thinking. The use of other approaches might have identified other phenomena as being central.

The implementation of the APHN initiative studied here consisted of distinct parts, and this should be kept in mind when considering the transferability of the findings to other implementation processes with less specific parts.

## Conclusions

The findings underline that managers in healthcare seem to have a substantial impact on how and to what extent different tasks are addressed and prioritized in top-down implementation processes. This has policy implications. The authors recognize that to achieve intended implementation outcomes, it is necessary to have an early and on-going dialogue about how the implementation task is perceived by the managers responsible for the implementation.

The present investigation is one of few studies that has built on the TEF model. Clearly, repeated studies in other settings are needed to further test this model and expand our understanding of how managers interpret and construct an implementation task, and also outline the implications that those aspects have for the implementation process.

## Additional file

Additional file 1: Interview Guide. (DOCX 13 kb)

## Abbreviations

APH: Academic Primary Healthcare
APHN: Academic Primary Healthcare Network
PHC: Primary Healthcare Centre
TEF: Transforming Experience Framework
