# Supplementary material for: Leading top-down implementation processes: a qualitative study on the role of managers
Source: BMC Health Serv Res. 2018 Jul 18;18:562. doi: 10.1186/s12913-018-3360-y (PMC6052667; doi:10.1186/s12913-018-3360-y)
Supplement: Supplementary file 1 — Interview Guide. (DOCX 13 kb) [file 12913_2018_3360_MOESM1_ESM.docx]

**Additional File 1. Interview guide**

You are appointed as manager of an APH coordinating centre let´s start from there.

- How do you look upon your role as manager of “X” APH network?

From your point of view, do you see any difference in managing an APH network in relation to managing a regular primary healthcare centre?

Do you consider it to be one or two assignments (manager of an APH network, manager of a primary healthcare centre)?

- What do you think is required of you in your role as manager to lead the introduction of an APHN in a good way?
- How do you look at the coordinator's role and responsibilities in introducing an APH network?

How do you look at the coordinator's role and responsibilities in relation to your role as a manager?

Differences? Possibilities? Difficulties? Reflections? Challenges?

- How do you look upon your employees' roles and responsibilities when introducing APH?

What expectations and requirements do you have on them?

How do you look at your role in enabling employees to take their responsibility?

- How do you look upon your opportunities to exercise your role as a manager?

Support from your own manager?

Support from the management group?

Support from others?

- Based upon the three parts of an APHN (research, professional development, education), how do you look upon your role as a manager?

What is your responsibility in your role as manager?

Do you see any differences in your role based on the different parts of the assignment?

- What do you consider to be the most important thing in your role as a manager of an APHN?
- Is there anything else you would like to add regarding your role as manager when introducing “X” APH network?
